# 69 Large vessel vasculitis occurring after COVID-19 infection: 2 Takayasu cases

**DOI:** 10.1093/rheumatology/keac496.065

**Published:** 2022-10-07

**Authors:** Vafa Guliyeva

**Affiliations:** Istanbul University, Istanbul Faculty of Medicine Turkey

## Abstract

**Background:**

Takayasu arteritis (TA) is a rare, chronic, granulomatous large vessel vasculitis that affects the aorta, its main branches, and the pulmonary artery. Although the aetiology of TA is not known entirely, it is thought to be multifactorial. To date, cases have been reported after Mycobacterium tuberculosis, human immunodeficiency virus (HIV), and influenza infection. Cases of postinfectious vasculitis have been reported in the literature during the COVID-19 pandemic. Here, we present two patients diagnosed with TA after COVID-19 infection.

**Case 1:**

A thirteen-year-old female patient was admitted to the pediatric emergency department of our hospital with chest pain and syncope. His uncle had a COVID-19 infection 6 months ago and they stayed in the same house during the infection.

On physical examination, respiratory sounds were less audible in the left lung baseline. The blood pressure was measured 128/71 mmHg in the right arm, 122/72 mmHg in the left arm, 106/67 mmHg in the right leg, and 107/74 mmHg in the left leg. Peripheral pulses were weaker in the lower extremities than in the upper extremities.

High acute phase reactants and anaemia were detected in the laboratory. (Table 1) Anti-ds DNA was positive, and anti-neutrophil cytoplasmic antibody (cANCA) was negative. Infection screening was negative.

In thorax and abdomen computed tomography (CT), 20 mm pericardium and 10 mm pleural effusion and mural thickening along the aorta of ∼20–25 cm from the descending thoracic aorta to the suprarenal level in the proximal 2 cm of the left subclavian artery and mild narrowing of the celiac and superior mesenteric artery orifices were detected. The patient was diagnosed with TA. She was treated with intravenous 30 mg/kg/day methylprednisolone for 3 days. The patient was discharged with oral prednisolone, methotrexate, and anti-TNF therapy.

**Case 2:**

A 17-year-old female patient was admitted to our center with complaints of back pain for 2 months, dry cough, weight loss (3 kg in 2 months), and fever for 2 days. It was learned that the patient had a COVID-19 infection 5 months ago.

On physical examination, blood pressure was measured 94/59 mmHg in the left arm, 103/60 mmHg in the right arm, 125/77 mmHg in the left leg, and 132/80 mmHg in the right leg. Peripheral pulses were weaker in the lower extremities compared with the upper ones. There was a 1/6 systolic murmur in the aortic focus. Other system examination was normal.

Laboratory tests revealed elevated acute phase reactants, thrombocytosis, and anaemia. (Table 1) Positron emission tomography-CT (PET-CT) revealed increased FDG uptake in both common carotid, aortic arch, ascending and descending aortic walls. In MR angiography, localized enlargement and stenosis of the aortic arch, wide ascending aorta, stenosis in the left subclavian artery, and the origin level of the left renal artery, and then a slight enlargement was observed. Based on the present findings, the patient was diagnosed with TA. Infection screening was negative. The patient was given 30 mg/kg/day intravenous methylprednisolone for 3 days and continued with oral prednisolone, methotrexate, and etanercept.

**Discussion:**

Timely diagnosis and treatment in TA is very important in preventing irreversible vascular damage resulting in ischaemia of vital organs. It should be kept in mind that TA and other vasculitides may occur after COVID-19 infection.

